# Protease induced plasticity: matrix metalloproteinase-1 promotes neurostructural changes through activation of protease activated receptor 1

**DOI:** 10.1038/srep35497

**Published:** 2016-10-20

**Authors:** Megan Allen, Suhasini Ghosh, Gerard P. Ahern, Sonia Villapol, Kathleen A. Maguire-Zeiss, Katherine Conant

**Affiliations:** 1 Interdisciplinary Program in Neuroscience, Georgetown University, Washington, DC, USA; 2 Department of Neuroscience, Georgetown University Medical Center, Washington, DC, USA; 3 Department of Pharmacology and Physiology, Georgetown University Medical Center, Washington, DC, USA.; 4 Department of Biology, Georgetown University, Washington, DC, USA.

## Abstract

Matrix metalloproteinases (MMPs) are a family of secreted endopeptidases expressed by neurons and glia. Regulated MMP activity contributes to physiological synaptic plasticity, while dysregulated activity can stimulate injury. Disentangling the role individual MMPs play in synaptic plasticity is difficult due to overlapping structure and function as well as cell-type specific expression. Here, we develop a novel system to investigate the selective overexpression of a single MMP driven by GFAP expressing cells *in vivo.* We show that MMP-1 induces cellular and behavioral phenotypes consistent with enhanced signaling through the G-protein coupled protease activated receptor 1 (PAR1). Application of exogenous MMP-1, *in vitro*, stimulates PAR1 dependent increases in intracellular Ca^2+^ concentration and dendritic arborization. Overexpression of MMP-1, *in vivo*, increases dendritic complexity and induces biochemical and behavioral endpoints consistent with increased GPCR signaling. These data are exciting because we demonstrate that an astrocyte-derived protease can influence neuronal plasticity through an extracellular matrix independent mechanism.

Matrix metalloproteinases (MMPs) are a family of zinc-dependent endopeptidases that remodel synaptic circuits and extracellular matrix (ECM) proteins through proteolytic processing. Regulated MMP activity contributes to physiological synaptic plasticity, while dysregulated activity stimulates neuronal injury^1^,^2^. In physiological plasticity, MMPs have traditionally been shown to influence the remodeling and growth of neuronal cellular elements through proteolytic cleavage of ECM proteins^3^,^4^. This generates soluble integrin binding ligands as well as a permissive environment for reorganization^5^,^6^,^7^. At the cellular level, dendrites or branched neuronal projections structurally change shape in response to extrinsic and intrinsic stimuli^8^. Dendritic growth and remodeling impacts the number of synaptic connections, which are crucial for ongoing cognitive processes, such as learning and memory^9^. Additionally, recent evidence that suggests astrocyte-derived factors greatly affect neuronal morphology^10^. In fact, a single astrocyte in the mouse cortex physically contacts several neuronal cell bodies, up to 600 dendrites, and approximately 100,000 synapses^11^,^12^. Notably, astrocytes secrete specific MMP family members in response to neuronal activity or pro-inflammatory stimuli^13^,^14^. Yet, very little is known about the role specific astrocyte-derived MMPs play in neuronal plasticity. Addressing this question is important to the field because it may uncover therapeutic targets for several disorders associated with increased MMP activity, astrocyte activation, and synaptic deficits including autism, fragile X syndrome, and Alzheimer’s disease^15^,^16^.

Over 20 distinct MMPs exist in the mammalian system, which are grouped according to structure and substrate preference, and then loosely grouped into families. Though several family members are expressed in the brain, including MMP-1, -2, -3, -9, and -13, many reports focus on the gelatinase, MMP-9, as a mediator of synaptic structure and function. These results do not, however, rule out a critical contribution from other MMP family members for which detection tools are less widely available^17^. In particular, few studies examine MMP-1 in the brain despite its expression by activated glia and its ability to act on non-ECM proteins. Our group previously reported that primary astrocytes, derived from human fetal brain, secrete abundant amounts of MMP-1 when activated by the pro-inflammatory cytokine IL-1β^14^. MMP-1 is a potent agonist for the non-ECM protein, protease activated receptor 1 (PAR1)^18^, a G-protein coupled receptor (GPCR) expressed on astrocytes and neurons in the hippocampus and cortex^19^,^20^,^21^. Further, activation of PAR1 plays a critical role in long-term potentiation, a form synaptic plasticity where synapses are strengthened through coordinated activity between neurons^19^,^22^,^23^. Receptor activation occurs through cleavage of a specific N-terminal domain, which uncovers a tethered signaling ligand^24^. MMP-1 is an equipotent activator when compared to the well-described peripheral PAR1 activator, thrombin. However, equimolar concentrations of other MMPs, including MMP-2, -3, and -7, do not activate downstream signaling events^18^.

Because PAR1 plays a critical role in activity dependent synaptic plasticity and MMP-1 is a potent agonist, we ask here whether select overexpression of MMP-1 is sufficient to induce cellular and behavioral phenotypes consistent with altered plasticity in a PAR1-dependent manner. To address this question, we use primary cell culture and a novel transgenic mouse that overexpresses human MMP-1 (hMMP-1 Tg) under the control of an astrocyte specific promoter, glial fibrillary acidic protein (GFAP). Importantly, human MMP-1 activates mouse PAR1^25^. Using a combination of approaches we report application of human recombinant MMP-1 (hRecMMP-1) to primary cultures enriched for neurons increases dendritic complexity and Ca^2+^ flux, which is reversed upon PAR1 genetic deletion or pharmacologic inhibition. Additionally, overexpression of astrocyte-derived hMMP-1 *in vivo* induces an increase in brain myo-Inositol levels, a marker of glial activation, as well as increases in dendritic complexity and spine density. hMMP-1 Tg animals also exhibit several behavioral phenotypes including alterations in anxiety and deficits in sociability and learning and memory. Excitingly, these findings suggest MMP-1 participates in circuit reorganization, and does so, in part, through a novel ECM-independent mechanism. Furthermore, this study expands our understanding of mechanisms through which proteolysis contributes physiological plasticity and suggests that the use of PAR antagonists may target aberrant plasticity.

## Results

### hRecMMP-1 application increases dendritic tree complexity as well as intracellular Ca^2+^ in primary culture

**Figure 1:**
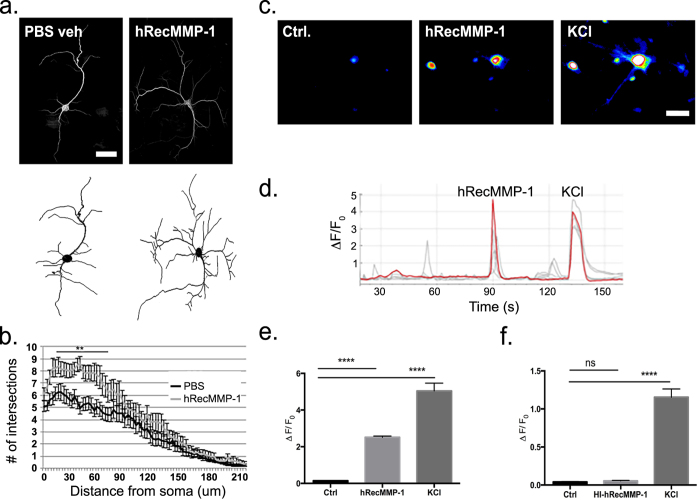
Application of hRecMMP-1 increases dendritic tree complexity as well as intracellular Ca^2+^ in primary culture. Immunocytochemical (ICC) labeling of hippocampal neuronal cytoskeleton with MAP2 antibody was performed to visualize cell morphology of neurons at DIV14, and representative images are shown in **(a)**: PBS vehicle control and hRecMMP-1 (8 nM) treated (scale bar = 25 μm). Digitized traces used for Sholl analysis for each corresponding neuron are shown below ICC images. Results from Sholl analysis reveal hRecMMP-1 treatment significantly increases the number of intersections at all distances from 9 μm–72 μm away from soma (PBS Veh n = 20 neurons, hRecMMP-1 n = 20 neurons; ANOVA with Bonferroni’s multiple comparisons post-test, **p value ≤ 0.01) **(b)**. To investigate Ca^2+^ flux, live cell imaging was performed on cultures enriched for neurons at DIV18. Representative Fluo-4 AM fluorescent images captured during application of control buffer, 40 nM hRecMMP-1, and 50 mM KCl are shown in **(c)**. Individual Ca^2+^ traces for each neuron from one representative experiment are shown in **(d)** with a single neuronal response highlighted in red. We quantify peak ΔF/F_0_ Ca^2+^ responses in **(e)** (n = 297 cells per condition; mean, ±SEM as follows: Ctrl 0.15 ± 0.01, hRecMMP-1 2.51 ± 0.06, KCl 5.05 ± 0.43; ANOVA with Bonferroni’s multiple comparisons post tests, ****p value ≤ 0.0001). Heat inactivated hRecMMP-1 (HI-hRecMMP-1, 40 nM) was administered in addition to control buffer and 50 mM KCl, and quantified peak ΔF/F_0_ Ca^2+^ responses are shown in **(f)** (n = 60 cells per condition; mean, ±SEM as follows: Ctrl 0.04 ± 0.01, HI-hRecMMP-1 0.06 ± 0.01, KCl 1.15 ± 0.11; ANOVA with Bonferroni’s multiple comparisons post-tests, significant *p* value ≤ 0.0001 and non-significant *p* value ≥ 0.05).

Astroglial membranes surround axon to dendrite synapses in the hippocampus^26^. Given this close physical proximity, researchers have long hypothesized that astrocyte-derived factors possess the capacity to influence dendritic spine structure and function. Astrocytes secrete high levels of proteolytic MMP enzymes^14^, yet very little is known about the neuronal structural changes following exposure to MMP-1. To address this question, we applied hRecMMP-1 (8 nM) to hippocampal neuron-enriched cultures and investigated differences in dendritic tree morphology at DIV14 using Sholl analysis. Representative images of neurons treated with PBS vehicle control or hRecMMP-1 and immunofluorescently labeled with MAP2 antibody are shown in (Fig. 1a, top). Images were then digitized with NeuronJ and Neuronstudio software (Fig. 1a, bottom) and analyzed with the semiautomated open source Bonfire MATLAB program^27^. hRecMMP-1 treatment significantly increases the number of dendritic crossings from 9 μm to 72 μm away from the cell soma (Fig. 1b) (*PBS veh n* = *20*, *hRecMMP-1 n* = *20*, *ANOVA with Bonferroni’s multiple comparisons post-tests*, *p value* ≤ *0.01*). These results suggest that application of hRecMMP-1 stimulates a more complex dendritic arrangement within 100 μm of the cell soma.

Ca^2+^ influx regulates dendritic patterning and previous evidence has shown that increases in intracellular Ca^2+^ activate transcription factors, including CAMKIV, which play a role in overall dendritic growth and patterning^28^,^29^. Locally, Ca^2+^ also affects dendritic stability by acting on proteins that regulate the rearrangement of actin and microtubules^30^,^31^. To investigate whether hRecMMP-1 treatment increased Ca^2+^ flux, we performed live cell Ca^2+^ imaging on primary cultures enriched for hippocampal neurons at DIV18 that were incubated with a fluorescent Ca^2+^ indicator dye (1 μM, Fluo-4 AM). After capture of a long baseline, cells were treated with 40 nM hRecMMP-1 and then 50 mM KCl. KCl quickly depolarizes neurons and permits flux of Ca^2+^ into the cell. This acted as a positive control to ensure cells in culture were alive and had the capacity to demonstrate Ca^2+^ flux. Representative Fluo-4 AM fluorescent images captured during application of control buffer, 40 nM hRecMMP-1, and 50 mM KCl are presented in Fig. 1c. Ca^2+^ traces for individual neurons from one experiment are shown in Fig. 1d and a representative trace is highlighted in red. The mean peak of ΔF/F_0_ Ca^2+^ responses is quantified Fig. 1e (*n* = *297 cells per condition; mean* ± *SEM from 7 experiments as follows: Ctrl 0.15* ± *0.01*, *hRecMMP-1 2.51* ± *0.06*, *KCl 5.05* ± *0.43*; *ANOVA with Bonferroni’s multiple comparisons post tests*, ****p value ≤ *0.0001*). Our results show that 84% of KCl responsive cells also respond to hRecMMP-1 with a sharp Ca^2+^ flux whereas 16% of KCl responsive cells did not respond to hRecMMP-1 treatment. To ensure that cells were responding to enzymatically active hRecMMP-1 with an increase in Ca^2+^ flux, heat inactivated hRecMMP-1 (HI-hRecMMP-1, 40 nM) was administered in addition to control buffer and KCl. These results are quantified in Fig. 1f (*n* = *60 cells per condition; mean* ± *SEM from 2 experiments as follows: Ctrl 0.04* ± *0.01*, *HI-hRecMMP-1 0.06* ± *0.01*, *KCl 1.15* ± *0.11; ANOVA with Bonferroni’s multiple comparisons post-tests*, significant ****p value ≤ *0.0001 and non-significant p value* ≥ *0.05*). Analysis of the non-neuronal cell population in cultures reveals that 10.86% of KCl non-responsive cells responded to hRecMMP-1 stimulation (n = 5 experiments, 66 cells out of 608 responded to hRecMMP-1 but not KCl).

### Generation and characterization human MMP-1 transgenic mouse (hMMP-1 Tg)

**Figure 2 f2:**
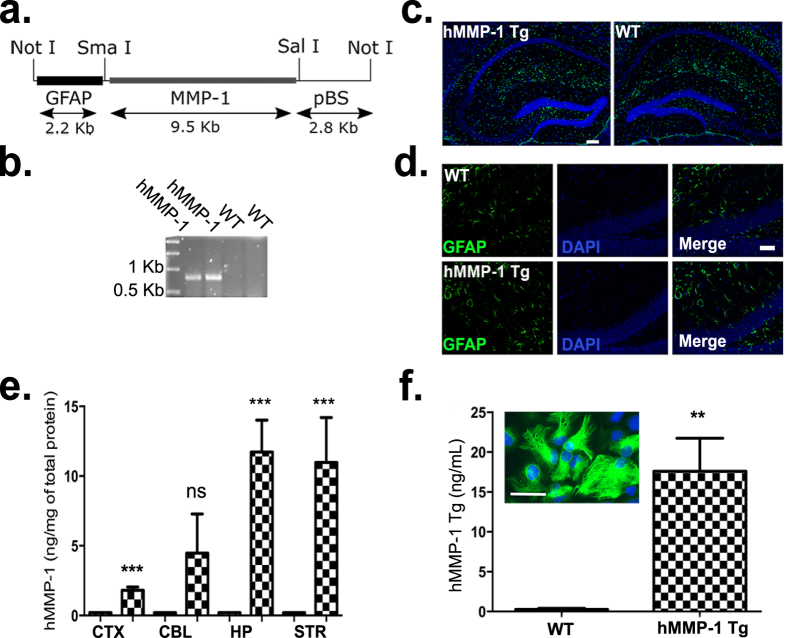
Generation and characterization of human MMP-1 transgenic mouse (hMMP-1 Tg). A schematic diagram of the construct used for pronuclear injections is shown in **(a)**. The murine GFAP promoter drives human MMP-1 expression in the transgenic model. To verify transgene presence, hMMP-1 was amplified by genomic PCR and a representative result is shown in **(b)**. GFAP immunofluorescent reactivity in WT and hMMP-1 Tg brains are shown in (**c**,**d**) GFAP (Green), Dapi (blue). Mean hMMP-1 protein levels (ng/mg of total protein) in discrete brain regions were measured by ELISA and are quantified in **(e)**. hMMP-1 levels in WT (n = 7) brain regions are not detected. Mean and ±SEM for hMMP-1 Tg (n = 8) brain regions: Cortex (CTX) 1.18, ±0.23; Cerebellum (CBL) 4.47, ±2.80; Hippocampus (HP) 11.71, ±2.29; Striatum (STR) 10.96, ±3.22 (Student’s t-test; ***p value ≤ 0.001, **p value ≤ 0.01, ns denotes a *p* value > 0.05). To ensure that astrocytes secrete hMMP-1, supernatants from primary astrocyte cultures were tested for the presence of hMMP1 by ELISA in **(f)** (WT n = 4, levels not detected; hMMP1 Tg n = 4, hMMP-1 mean, ±SEM: 17.60 ng/mL, ±4.14; Student’s t-test, **p value = 0.005. The inset contains a representative image of cultured cortical astrocytes; GFAP immunocytochemistry confirmed 95% of the cells were GFAP positive. Scale bars = 200 μm (**c**), 100 μm (**d**), 25 μm (**f**).

To investigate whether MMP-1 induces neuronal structural changes *in vivo*, we engineered a novel mouse to overexpress hMMP-1 driven by GFAP. Neurostructural changes in this context may have consequences for learning and memory processes. In our system, the GFAP promoter controls human MMP-1 overexpression (schematic in Fig. 2a). Genomic PCR analysis confirms human MMP-1 (hMMP-1) expression in transgenic animals but not wild-type (WT) littermates (Fig. 2b). Initial PCR products were sequenced and matched against the National Center for Biotechnology Information’s (NCBI) database for hMMP-1. We next find no significant difference in pixel intensity of GFAP immunofluorescent reactivity between WT and hMMP-1 Tg brains in hippocampal region (*images shown in* Fig. 2c,d; *WT and hMMP-1 Tg n* = *2*, *mean pixel intensity* (*a.u.*), ±*SEM as follows: WT 7.94* ± *0.42*, *hMMP-1 Tg 8.21* ± *0.42*). We also quantify hMMP-1 protein in discrete brain regions from hMMP-1 Tg mice by ELISA (Fig. 2e). hMMP-1 is significantly higher in the cortex, hippocampus, and striatum, but not cerebellum of transgenic animals when compared to WT littermate controls (*WT n* = *7*, *hMMP-1 Tg n* *=* *8; Mean hMMP-1 protein levels* (*ng/mg of total protein*) *in discrete brain regions with* ±*SEM were as follows: cortex 1.18*, ±*0.23. p value* = *0.00001; cerebellum 4.47*, ±*2.80*, *p value* = *0.162; hippocampus 11.71*, ±*2.29*, *p value* = *0.0005; striatum 10.96*, ±*3.22*, *p value* = *0.007*). Lastly, to determine whether astrocytes secrete the overexpressed protein, we measure supernatants from primary astrocyte cultures derived from cortices extracted from WT and hMMP-1 Tg for the presence of hMMP-1. A representative image of GFAP-positive primary astrocytes is shown in Fig. 2f (inset). hMMP-1 ELISA analysis of astrocyte culture media show that hMMP-1 Tg positive astrocytes do release hMMP-1 while this protein was not detected in WT astrocyte supernatants (Fig. 2f; *WT n* = *4*, *levels not detected; hMMP-1 Tg n* = *4*, *hMMP-1 mean*, *SEM: 17.60* *ng/mL of supernatant*, ±*4.14; Student’s t-test*, **p value = *0.005*). We confirm that the secreted hMMP-1 is active in supernatants of cultures containing both neurons and astrocytes using a fluorogenic based substrate assay. While activity in concentrated (40X) WT supernatants (duplicate samples from 2 replicate cultures) is not detected, activity in concentrated (40X) hMMP-1 Tg supernatants (duplicate samples from three replicate cultures) is equivalent to 3.75 ± 1.28 ng of recombinant hMMP-1. Results are significant by Student’s t-test (*p value = 0.04).

### Concurrent expression of hMMP-1 mRNA and GFAP protein *in situ*

**Figure 3 f3:**
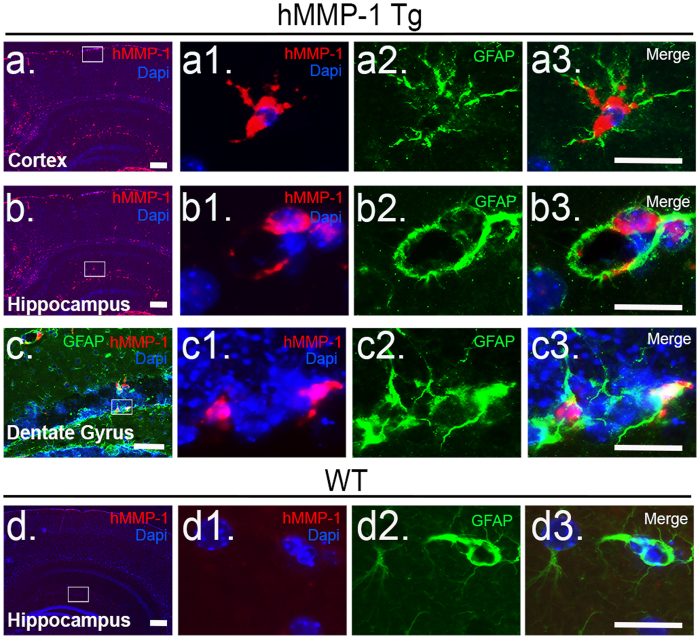
Concurrent expression of hMMP-1 mRNA and GFAP protein. Brain coronal sections were assayed for the presence of hMMP-1 mRNA (red), GFAP protein (green), and DAPI nuclei (blue) in hMMP-1 Tg and WT animals. We highlight several regions of interest **(a–d)**. Low magnification images show the distribution of cells that concurrently express hMMP-1 mRNA as well as GFAP protein in hMMP-1 Tg mouse brain **(a–c)**. White boxes **(a–d)** indicate the region of interest for high power views. These high magnification views show cortical GFAP positive astrocytes with a hMMP-1 mRNA signal (a1–a3), and a stereotypical astrocytic foot process surrounding a blood vessel in the hippocampus that espressses both GFAP and hMMP-1 mRNA (b1–b3). Lastly, GFAP positive cells expressing hMMP-1 mRNA in the hippocampal dentate gyrus are also shown (c1–c3). Notably, hMMP-1 mRNA is not present in WT littermate control brain **(d)**, GFAP positive cells lacking hMMP-1 mRNA are shown in (d1–d3). Scale bars: 200 μm (**a**,**b**,**d**), 50 μm (**c**), and 20 μm (a1–a3, b1–b3, c1–c3, d1–d3).

To verify that GFAP positive cells express the transgene, we explored the colocalization of hMMP-1 mRNA using fluorescent *in situ* hybridization (FISH) combined with GFAP immunofluorescent chemistry (IFC) (Fig. 3). We find that hMMP-1 mRNA is restricted to GFAP positive cells in hMMP-1 Tg animals but not WT littermate controls. A low magnification image taken from hMMP-1 Tg brain with an area of interest in cortex and hippocampus highlighted in white (Fig. 3a,b, respectively). Higher magnification views for cortical and hippocampal areas of interest are shown in Fig. 3a1–a3, and Fig. 3b1–b3, [Fig f2], [Fig f3], respectively. Cell morphology indicates that double positive cells are most likely astrocytes. Further analysis reveals that cells in the dentate granule cell layer (Fig. 3c, low magnification) are positive for both GFAP and hMMP-1 mRNA (Fig. 3c1–c3, [Fig f2], [Fig f3], high magnification). Based on localization in this layer, double positive cells could represent astrocytes and/or neural progenitor cells^32^. Lastly, a GFAP positive cell lacking hMMP-1 mRNA in WT littermate control brain is presented for comparison in Fig. 3d (low magnification). Higher magnification views for the WT hippocampal region of interest, outlined in white, are shown in Fig. 3d1–d3, [Fig f2], [Fig f3]. Notably, results from quantitative real-time PCR (qRT-PCR) reveal mRNA for the murine orthologue of MMP-1, *Mmp-1a*, is undetectable in both WT and hMMP-1 Tg mouse brains (Supplementary Table 1). The primers detected *Mmp-1a* transcripts in our positive control (primary astrocytes activated with IL-1β). In addition, transcript levels of the functionally similar mouse MMP, *Mmp-13*, do not differ significantly between genotypes (Supplementary Table 1).

### hMMP1 Tg animals show changes in brain metabolites relevant to gliosis as determined by *in vivo* MRS imaging

**Figure 4:**
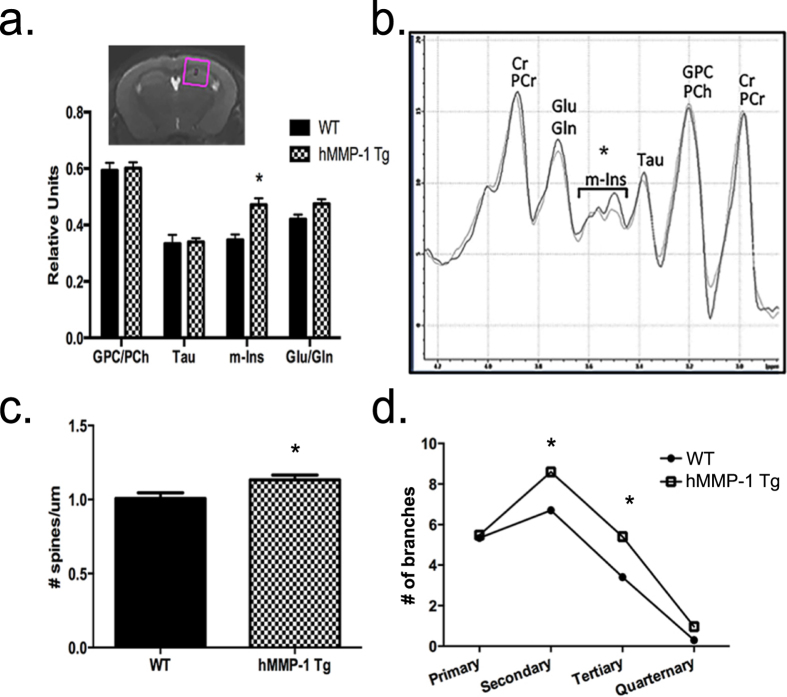
Increased levels of the glial activation marker, myo-inostiol (m-Ins), coincide with changes in neuronal morphology. We show a representative image of a mouse brain highlighting the region of interest analyzed for metabolite levels (a, inset, pink box) by *in vivo*^1^H MR. Comparisons in spectra from hMMP-1 Tg (n = 6) and WT (n = 6) were normalized to total creatine (**a**,**b**). (Mean, ±SEM are as follows: *Cho* WT 0.59, ±0.07, hMMP-1 Tg 0.60, ±0.05; *Tau* WT 0.33, ±0.08, hMMP-1 Tg 0.34, ±0.03; *myo-I* WT 0.35, ±0.04, hMMP-1 Tg 0.47, ±0.06; *Glu/Gln* WT 0.42, ±0.04, hMMP-1 Tg 0.48, ±0.04. *Statistics:* 2-way ANOVA with Bonferonni’s post test; *p* value ≤ 0.05. Abbreviations: Cr, creatine; PCr, phosphocreatine; Glu, glutamate; Gln, glutamine; m-Ins, myo-Inositol; Tau, taurine; GPC, glycerophosphocholine; PCH, phosphocholine). To examine neuronal morphology *in vivo*, the Golgi impregnation technique was performed. We find increased spine density on apical dendrites of CA1 hippocampal pyramidal cells in hMMP-1 Tg when compared to WT littermate controls (**c**) (WT n = 56 dendrites from 15 neurons per animal from 4 animals, excluded 4 dendrites because out of focus; hMMP1 Tg n = 80 dendrites from 16 neurons per animal from 5 animals; Mean followed by ±SEM: WT 1.01 ± 0.03, hMMP-1 Tg 1.13 ± 0.03; Student’s t-test *p value = 0.02). Also, hMMP-1 Tg animals exhibited a significantly higher number of secondary and tertiary dendrites from pyramidal neurons in layer IV/V of the somatosensory cortex (**d**) (hMMP-1 Tg n = 3 animals and WT n = 3 animals; mean followed by ±SEM: WT primary 5.33 ± 0.22, secondary 6.70 ± 0.41, tertiary 3.41 ± 0.63, quaternary 0.30 ± 0.14, hMMP-1 Tg primary 5.48 ± 0.26, secondary 8.60 ± 0.42, tertiary 5.41 ± 0.66, quaternary 0.96 ± 0.28; ANOVA, *p value ≤ 0.05).

In our model, astrocytes secrete hMMP-1, which could influence varied cell populations. To investigate the effects of hMMP-1 on the living brain, we examined specific brain metabolites including myo-Inositol because these levels increase in the setting of astrocyte and/or microglial activation^33^. To evaluate regional changes in metabolites, we use *in vivo* MRS to analyze both hippocampal and cortical regions of hMMP-1 Tg mice and WT littermate controls (region of interest depicted in Fig. 4a inset image). We find a significantly higher level of myo-Inositol in hMMP-1 Tg brains when compared to WT littermate controls. Genotype does not significantly alter levels of other metabolites measured in this study. Metabolite levels from *in vivo*^1^H MR spectra analysis, are normalized to total creatine (Fig. 4a,b) (*Mean*, ±*SEM are as follows: Cho WT 0.59*, ±*0.07*, *hMMP-1 Tg 0.60*, ±*0.05; Tau WT 0.33*, ±*0.08*, *hMMP-1 Tg 0.34*, ±*0.03; myo-I WT 0.35*, ±*0.04*, *hMMP-1 Tg 0.47*, ±*0.06; Glu/Gln WT 0.42*, ±*0.04*, *hMMP-1 Tg 0.48*, ±*0.04. Statistics: ANOVA with Bonferonni multiple comparisons post-tests; p value* ≤ *0.05*). These results suggest that hMMP-1 secreted from GFAP positive cells may be acting on astrocytes and/or microglia.

### hMMP-1 transgenic animals display dendritic changes indicative of structural remodeling at the synapse

Results from our *in vitro* Sholl assays suggest hRecMMP-1 stimulation induces morphological changes in the dendritic arbor of hippocampal neurons. To investigate whether astrocyte-derived hMMP-1 induces change in neuronal structure *in vivo*, we performed a Golgi impregnation protocol on whole brains from MMP-1 Tg and WT littermate controls to visualize dendritic arbors as well as dendritic spines. hMMP-1 Tg animals have a significant, but small, increase in spine density in apical dendrites of CA1 pyramidal neurons (Fig. 4c; *average spines/10 μm dendritic section with* ±*SEM: WT 1.01*, ±*0.03*, *n* = *56 dendrites; hMMP-1 Tg 1.13*, ± *0.03*, *n* = 80 dendrites; Student’s t-test *p value = *0.02*). hMMP-1 Tg also display enhanced dendritic complexity in secondary and tertiary dendritic branches in pyramidal neurons of layer IV/V of somatosensory cortex (Fig. 4d; *hMMP-1 Tg and WT n* = *3 animals*, *27 neurons; mean followed by* ±*SEM: WT primary 5.33* ± *0.22*, *secondary 6.70* ± *0.41*, *tertiary 3.41* ± *0.63*, *quaternary 0.30* ± *0.14; hMMP-1 Tg primary 5.48* ± *0.26*, *secondary 8.60* ± *0.42*, *tertiary 5.41* ± *0.66*, *quaternary 0.96* ± *0.28; ANOVA*, *p value ≤ *0.05*). These results suggest that overexpression of hMMP-1 in GFAP positive cells stimulates an increase in spine density as well as more complex dendritic branching *in vivo*.

### hMMP-1 transgenic animals displayed deficits in behaviors associated with synaptic plasticity

**Figure 5:**
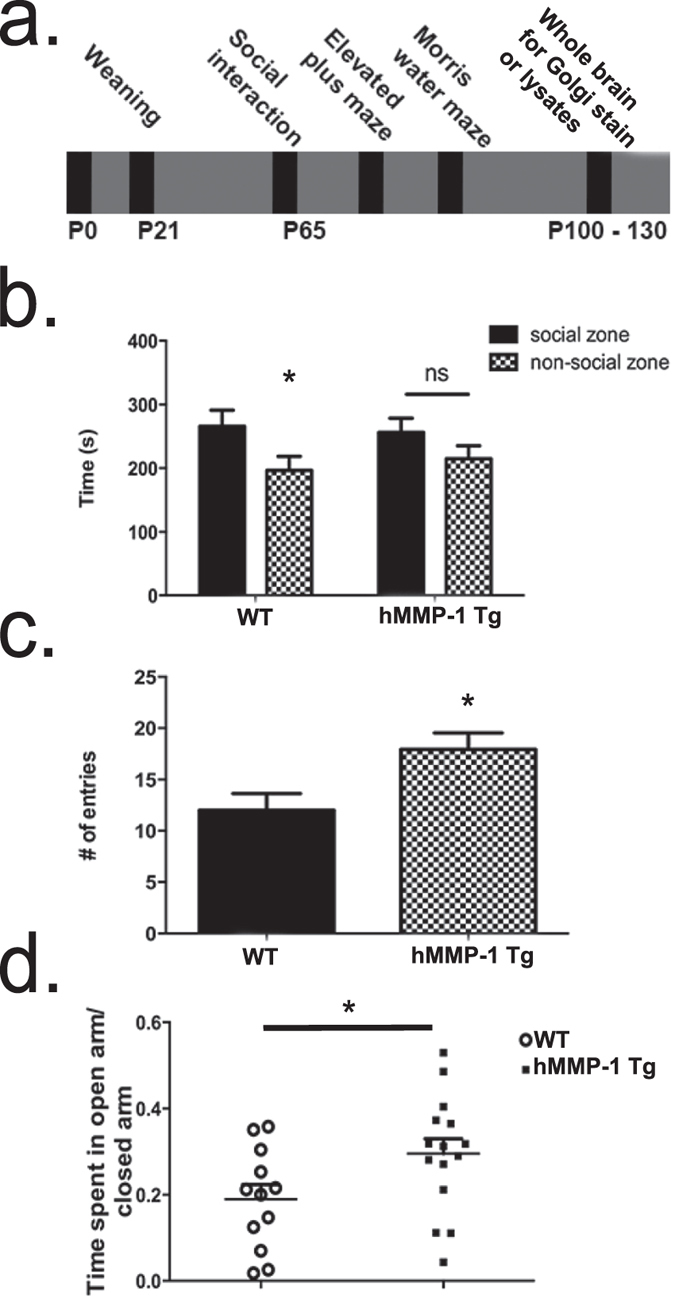
hMMP-1 Tg animals exhibit deficits in behaviors associated with synaptic plasticity. A schematic diagram **(a)** of an experimental timeline showing behavioral tests performed in order of low to high stress. Sociability testing reveals hMMP-1 Tg show no significant preference for social stimulus as compared to WT littermate controls **(b)** (WT n = 10, Student’s t-test *p value = 0.05; hMMP-1 Tg n = 14, Student’s t-test *p* value = 0.18), elevated plus maze results show decreased anxiety as noted by significantly more entries into the open arm region of the apparatus **(c)** (WT n = 12, hMMP-1 Tg n = 15; Student’s t-test *p value = 0.01) as well as **(d)** time spent in the open arm relative to the total time of the test (Student’s t-test *p value = 0.04).

Because structural changes in dendritic complexity and spine number have consequences for cognitive processes, we next investigated deficits in plasticity-related behaviors. We performed a battery of behavioral assays pertinent to cognitive processes that require activity dependent synaptic plasticity, including sociability, anxiety, and hippocampal learning and memory. Notably, disruptions in all three of these behavioral domains have been reported animals models of autism and autism-related disorders, which have altered spine density and dendritic complexity^34^. In our tests, behavioral assays were performed in order from least to most stressful (refer to Fig. 5a for experimental timeline). During sociability testing, habituated mice were able to freely explore a three-chambered box that contained a social stimulus at one end and a non-social stimulus at the other. Results show that hMMP-1 Tg mice display decreased sociability (Fig. 5b). WT animals spent significantly more time in the social quadrant (*WT n* = *10*, Student’s t-test *p value = *0.05*, *266* *s* ±*25* *s compared to 196* *s* ±*22*). In contrast, hMMP-1 Tg animals showed no preference toward the social quadrant, (*hMMP-1 Tg n* = *14*, *Student’s t-test p value* = *0.18*, *256* *s* ±*22* *s compared to 215* *s* ±*20* *s*).

Next, we examined performance on the elevated plus maze (EPM). This assay has been pharmacologically proven to measure anxiety in mice^35^. We find that hMMP-1 Tg animals enter the open arm of the maze more times that WT littermate controls (Fig. 5c; *WT n* = *12*, *hMMP-1 Tg n* = *15; 18* ± *1.70 entries compared to 12* ± *1.50 entries*, Student’s t-test *p value = *0.01*). Additionally, hMMP-1 Tg animals spent significantly more time in the open quadrant than WT controls as shown in Fig. 5d (*88* *s* ±*10 compared to 57* *s* ±*10*, Student’s t-test *p value = *0.04*).

**Figure 6:**
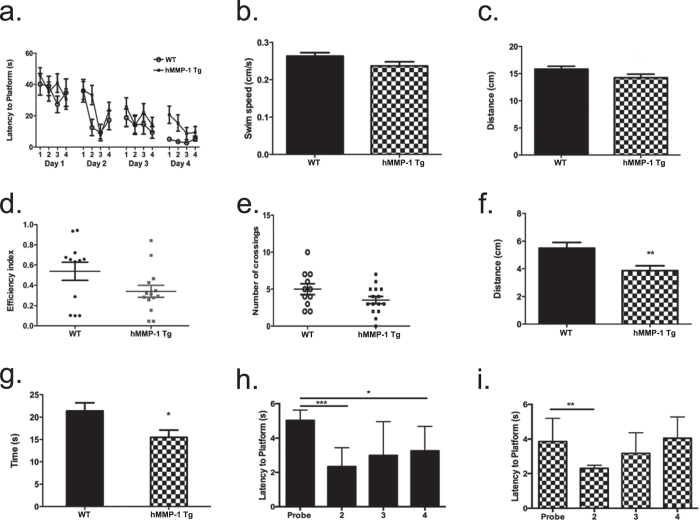
hMMP-1 Tg animals display deficits in learning and memory on the Morris water maze. Results from Morris water maze testing (WT n = 11; hMMP-1 Tg n = 15) are presented in **(a–i)**. **(a)** We find no statistical differences in the latency to locate the hidden platform during training **(a)** (4 trials administered for 4 consecutive days) suggesting both groups were able to perform task and indicating an appropriate level of training (2-way ANOVA *p* value > 0.05). On the fifth day, the hidden platform was removed and several endpoints were measured. Swim speed **(b)** and total distance travelled **(c)** during the probe trial are not significantly altered between the WT and hMMP-1 Tg suggesting that motor impairments do not account for differences observed during probe trial (Student’s t-test *p* value > 0.05). hMMP-1 Tg exhibited decreased path efficiency (**d)** Student’s t-test *p* value = 0.07) as well as crossings over the area that formerly housed the platform **(e)** Student’s t-test *p* value = 0.09). Notably, hMMP-1 Tg animals travel significantly less in the quadrant that formerly housed the invisible platform **(f)** (Student’s t-test **p value = 0.01) and spend less time in this quadrant **(g)** (Student’s t-test *p value = 0.03) when compared to WT littermate controls. Times spent in all quadrants during the probe trial, which tests reference memory, are presented for WT animals in **(h)** and hMMP-1 Tg animals in **(i)**.

To test hippocampal-dependent spatial memory deficits, we utilized the Morris water maze task (WT n = 11; hMMP-1 Tg n = 15). During the training phase, animals were trained to find an invisible platform in a pool of opaque water for four trials over four days. The latency to platform was recorded. On the fifth day (probe trial), the invisible platform was removed and time and distance traveled in the quadrant that previously housed the platform was recorded. Notably, both groups were able to perform the task as no significant differences were observed during training, which indicates appropriate training efficiency (Fig. 6a; *ANOVA*, *p value* ≥ *0.05*). Additionally, we report in Fig. 6b,c that both swim speed (*WT 0.26* *cm/s* ±*0.01 compared to hMMP-1 Tg 0.24* *cm/s* ±*0.01*) and total distance traveled (*WT 15.82* *m* ±*0.53 compared to hMMP-1 Tg 14.23* *m* ±*0.66*) during the probe trial did not differ between groups (*Student’s t-test p value* ≥ *0.05*). These three control measures suggest that hMMP-1 Tg animals do not display motor impairments and/or task induced anxiety that would prevent accurate interpretation of the experimental results. We find a trend toward decreased path efficiency (Fig. 6d; *WT 0.54*, ±*0.09 compared to hMMP-1 Tg 0.34*, ±*0.06; Student’s t-test p value* = *0.07*) and crossings by the hMMP-1 Tgs in the area that formerly housed the platform (Fig. 5e; *WT 5.0*, ±*0.73 compared to hMMP-1 Tg 3.5*, ±*0.5; Student’s t-test p value* = *0.09*).

Consistent with these results, data from the probe trial, which is 24 hours after the last training session and measures memory^36^, reveal hMMP-1 Tg animals travel significantly less distance in the quadrant that formerly housed the hidden platform (Fig. 6f; *WT 5.49* *m* ±*0.35 compared to hMMP-1 Tg 3.86* *m* ±0.35; Student’s t-test *p value = *0.01*). As expected, hMMP-1 Tg animals also spend significantly less time in the quadrant that formerly housed the hidden platform (Fig. 6g; *WT 21.36* *s* ±*1.81 compared to hMMP-1 Tg 15.48* *s* ±*1.64; Student’s t-test p value* = *0.03*). Time spent in each quadrant on probe trial day is shown for WT (Fig. 6h; *probe quadrant: 5.03* *s* ±*0.60*, *quadrant 2: 2.34* *s* ±*0.32*, *quadrant 3: 2.99* *s* ±*0.57*, *quadrant 4: 3.25* *s* ±*0.41; ANOVA*, *p value* ≤ *0.05*) and hMMP-1 (Fig. 6i; *probe quadrant: 3.86* *s* ±*0.35*, *quadrant 2: 2.30* *s* ±*0.18*, *quadrant 3: 3.17* *s* ±*0.31*, *quadrant 4: 4.05* *s* ±*0.32; ANOVA*, *p value* ≤ *0.05*).

### Evidence supporting PAR1 activation in hMMP-1 Tg animals

**Figure 7:**
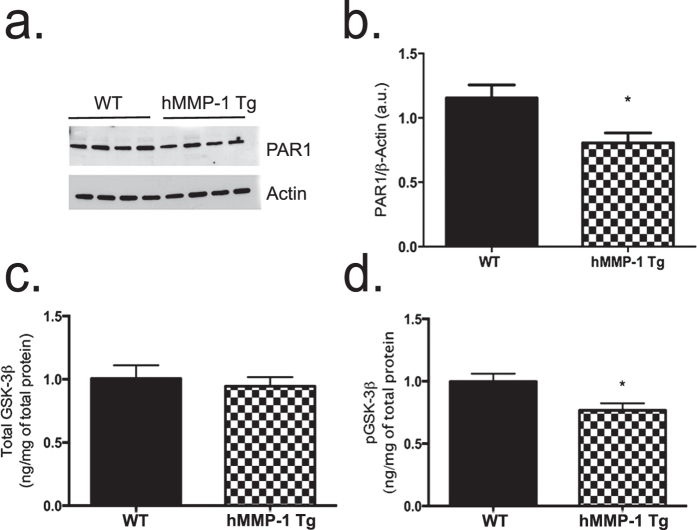
PAR1 is activated in hMMP-1 Tg animals. Western blot analysis shows PAR1 protein levels in cortical/hippocampal lysates. A representative image of the western blot is shown **(a).** Quantification with densitometry is shown in **(b)** (WT n = 4, hMMP-1 Tg n = 4; mean followed by ±SEM: WT 1.16 ± 0.10, hMMP-1 Tg 0.81 ± 0.10; Student’s t test, *p value = 0.03). Results in **(c,d)** show that GSK-3β activity is increased in mice that overexpress hMMP-1. We assayed hippocampal lysates and detected total GSK-3β and phospho-GSK-3β at serine 9 by ELISA. Decreased phosphorylation at serine 9 activates the kinase (Total GSK-3β: WT n = 17, hMMP-1 Tg n = 21, mean followed by ±SEM: WT 1.00 ± 0.10, hMMP-1 Tg 0.94 ± 0.07; Student’s t test, *p* value = 0.62; pGSK-3β: WT n = 14, hMMP-1 Tg n = 21, mean followed by ±SEM: WT 1.00 ± 0.06, hMMP-1 Tg 0.77 ± 0.05; Student’s t test, *p value = 0.01).

To address the question of increased PAR1 GPCR signaling in hMMP-1 Tg animals, we examined protein levels of PAR1 in cortical/hippocampal lysates. It has been shown that following activation (i.e. tethered ligand binding), PAR1 is rapidly internalized with only 25% trafficked back to the cell membrane^37^. Moreover, in a disease model with increased PAR1 signaling in the lung, receptor protein levels are decreased^38^. Consistent with receptor activation, PAR1 protein levels are significantly decreased in cortical/hippocampal lysates from hMMP-1 Tg when compared to WT littermate controls as shown by Western blot analyses (Fig. 7a,b; *WT n* = *4*, *hMMP-1 Tg n* = *4; mean followed by* ±*SEM: WT 1.16 a.u.* ±*0.10*, *hMMP-1 Tg 0.81 a.u.* ±*0.10; Student’s t test*, *p value = *0.03*).

PAR1 signaling may contribute to altered dendritic spine density through β-arrestin-mediated activation of GSK-3β^39^. GSK-3β activation through dephosphorylation of serine 9 has been linked to an increase in thin dendritic spines on hippocampal neurons and PAR1 signaling^40^,^41^. To determine whether GSK-3β activity is increased in the hMMP-1 Tg mice, we tested hippocampal lysates for serine 9 phosphorylated and total GSK-3β levels by ELISA. Results in Fig. 7c,d show that compared to WT mice, hMMP-1 Tg mice display no change in total GSK-3β protein but have a significant decrease in phosphorylated GSK-3β; indicative of increased GSK-3β activity with hMMP-1 overexpression^42^ (*Total GSK-3β: WT n* = *17*, *hMMP-1 Tg n* = *21*, *mean ng/mg of total protein followed by* ±*SEM: WT 1.00* ± *0.10*, *hMMP-1 Tg 0.94* ± *0.07; Student’s t test*, *p value* = *0.62; pGSK-3β: WT n* = *14*, *hMMP-1 Tg n* = *21*, *mean followed by* ±*SEM: WT 1.00* ± *0.06*, *hMMP-1 Tg 0.77* ± *0.05; Student’s t test*, *p value = *0.01*).

### Pharmacological inhibition and genetic deletion of PAR1 reverse hRecMMP-1 effects on neuronal morphology and Ca^2+^ flux

**Figure 8:**
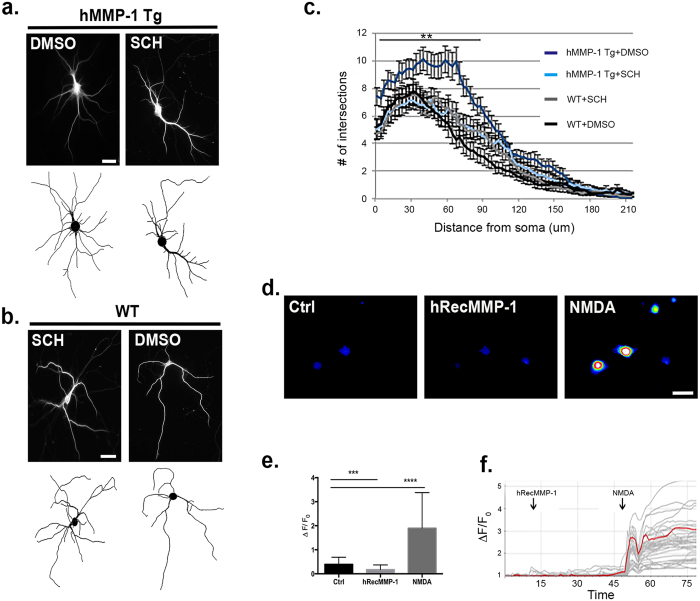
PAR1 inhibition and genetic deletion reverse MMP-1 induced effects on neuronal morphology and Ca^2+^ flux. Representative images from neurons cultured from hMMP-1 Tg and WT animals treated with DMSO vehicle control or a PAR1 inhibitor (SCH79797; SCH) after immunofluorescent labeling of neuronal cytoskeleton with MAP2 antibody at DIV14 **(a,b)**. The digitized trace used for Sholl analysis for each corresponding neuron is shown below. Results from Sholl analysis reveal that neurons cultured from hMMP-1 Tg animals have a significantly increased number of intersections from 0 μm to 90 μm from cell soma when compared to hMMP-1 Tg + SCH, WT + SCH, and WT + DMSO groups **(c)** (hMMP-1 Tg + DMSO n = 20 neurons, hMMP-1 Tg + SCH n = 20 neurons, WT + SCH n = 20 neurons, WT + DMSO n = 20 neurons; ANOVA with Dunnett’s post-tests, **p value ≤ 0.01). To measure Ca^2+^ flux, live cell calcium imaging was performed on cultures enriched for neurons derived from PAR1 KO pups at DIV18 **(d,e)**. We recorded during application of control, 40 nM hRecMMP-1, and 50 nM NMDA. Representative still images taken during application are shown in **(d).** The mean peak of ΔF/F_0_ Ca^2+^ responses from each cell is quantified in **(e)** (N = 65 cells per each condition, normalized mean, SEM: Ctrl 0.39 ± 0.04, hRecMMP-1 0.17 ± 0.03, NMDA 1.90 ± 0.22; ANOVA with Bonferroni’s multiple comparisons post tests, ***p value ≤ 0.001 and ****p value ≤ 0.0001). Calcium traces for individual neurons from one experiment are shown in gray and a representative trace is highlighted in red **(f)**.

The potential for MMPs to act on specific targets depends on several factors including substrate availability and proximity. MMP-1 is most active at the cell surface^43^ and is a potent agonist for cell surface PAR1. Interestingly, PAR1 has been found in synaptosomal preparations, which most likely includes PAR1 located on astrocytic processes that cradle synapses as well as pre- and post-synaptic neuronal membranes^44^. To interrogate the role the MMP-1/PAR1 signaling pathway plays in dendritic arborization, we derived neuron-enriched cultures from MMP-1 Tg and WT animals. These cells were treated with DMSO vehicle control or a PAR1 inhibitor (SCH79797; SCH). Subsequent analysis shows that neurons cultured from hMMP-1 Tg animals have significantly increased number of intersections from 0 μm to 90 μm from cell soma when compared to hMMP-1 Tg + SCH, WT + SCH, and WT + DMSO groups (Fig. 8a,b,c) (*MMP-1 Tg* + *DMSO n* = *20 neurons*, *MMP-1 Tg* + *SCH n* = *20 neurons*, *WT* + *SCH n* = *20 neurons*, *WT* + *DMSO n* = *20 neurons; ANOVA with Dunnett’s post-tests*, *p value* ≤ *0.01*). Our results suggest MMP-1 activation of PAR1 is responsible for the increased dendritic complexity because hMMP-1 Tg group treated with the PAR1 inhibitor do not significantly differ from WT neurons. Notably PAR1 inhibition does not affect the results of Sholl analysis in WT group suggesting aberrant over activation of the receptor as seen in the hMMP-1 Tg group is required for the morphological change.

To investigate whether the MMP-1/PAR1 axis is also involved in Ca^2+^ flux, which may influence dendritic arbor patterning, we performed live cell Ca^2+^ imaging with cultures enriched for neurons derived from PAR1 KO pups at DIV18. Representative images taken during the application of control, 40 nM hRecMMP-1, and 50 nM N-methyl-D-aspartate (NMDA) are shown in Fig. 8d. The mean peak of ΔF/F_0_ Ca^2+^ responses from each cell is quantified in Fig. 8e (*N* = *65 cells per each condition*, *normalized mean*, *SEM: Ctrl 0.39* ± *0.04*, *hRecMMP-1 0.17* ± *0.03*, *NMDA 1.90* ± *0.22; ANOVA with Bonferroni’s multiple comparisons post tests*, ***p value ≤ 0.001 and ****p value ≤ *0.0001*). NMDA was used as a positive control in these experiments as it quickly depolarizes neurons and permits flux of Ca^2+^ into the cell. Ca^2+^ traces for individual neurons from one experiment are shown in gray and a representative trace is highlighted in red (Fig. 8f). We find that genetic deletion of the GPCR PAR1 prevents the hRecMMP-1-mediated increase in Ca^2+^ in 95% of NMDA responsive cells. Five percent of NMDA responsive cells responded to treatment with an increase in Ca^2+^ suggesting that MMP-1 acts on a small subset of cells through a PAR1 independent pathway.

## Discussion

Our results demonstrate a novel mechanism through which brain-derived MMP-1 directs neuronal dendritic organization by activating the GPCR PAR1. Interestingly, this work also strengthens previous findings that show astrocytic factors affect structural remodeling, which underlie physiological synaptic plasticity. Studies in primary culture reveal that MMP-1-mediated PAR1 activation enhances the complexity of neuronal dendritic arbors and Ca^2+^ flux. Additionally, hMMP-1 Tg mice display increased spine density in CA1 hippocampal neurons and increased complexity of dendritic arbors in layer IV/V of somatosensory cortex. At the behavioral level, astrocyte-derived MMP-1 induces several phenotypes associated with altered synaptic plasticity including decreased anxiety and deficits in sociability and hippocampal dependent memory. Taken together, we show here that a single MMP driven by GFAP expression can influence neuronal plasticity at least in part through an ECM independent mechanism. Modulation of this axis could provide a therapeutic target for disorders in which astrocyte activation and elevated levels of MMPs are observed^45^.

Disentangling the role individual MMPs play in synaptic plasticity has been especially difficult for the field because these proteases share overlapping structure and function and activate one another. Indeed, redundancy in proteolytic processing of substrates is especially strong among functionally related MMPs. MMP-1 is a soluble collagenase named for its processing of extracellular fibrillar collagen and includes MMP-8 and -13 as sub-family members^46^. MMP-8 exhibits differences in function and expression profile when compared to MMP-1 and -13^47^. Results from real-time quantitative reverse transcription PCR (qRT-PCR) reveal mRNA transcripts for murine MMPs that are functionally similar to hMMP-1, *Mmp-1a and Mmp-13*, do not differ significantly in the brains of hMMP-1 Tg animals when compared to WT littermate controls. These results suggest they are not upregulated in our model, and thus unlikely contribute to differences reported herein.

Though our work is the first to focus on MMP-1 and neuroplasticity, it complements previous studies that implicate other MMP family members in structural and functional changes, including stromelysin (MMP-3) and gelatinases (MMP-2 and -9). For example, in Drosophila models, deletion of *mmp2* completely blocks dendrite reshaping attributed to local degradation of the basement membrane^48^,^49^. Similarly, in murine systems, deletion of *Mmp2* and *Mmp3* induce a reduction in dendritic arbor surrounding purkinje cells of the cerebellum and apical dendritic length in layer 5 pyramidal neurons of the visual cortex, respectively^50^,^51^. In addition, mature cortical neurons treated with a pan-metalloproteinase inhibitor show reduced neurite outgrowth^52^. These studies, although experimentally rigorous, do not focus on specific substrates nor do they evaluate the role of cell-type specific MMP expression. It has been hypothesized that pan-MMP processing of the ECM generates a permissive environment for the rearrangement of dendritic structures. MMPs liberate integrin binding laminin fragments and soluble adhesion molecule ectodomains, which have the potential to influence actin dependent structural plasticity^53^,^54^. Yet, the specific MMPs and mechanisms that are critical for these changes remain unclear. Complicating the interpretation of results even further is the use of inhibitors that target highly conserved catalytic domains across family members, and therefore lack specificity^55^.

Herein, we show pharmacologic inhibition of a specific substrate, PAR1, reverses increases in dendritic branching complexity in hippocampal neurons due to hMMP-1 overexpression. Likewise, genetic deletion of PAR1 prevents MMP-1 induced flux of intracellular Ca^2+^. Astrocyte-derived hMMP-1 increases branching complexity in layer IV/V neurons of the somatosensory cortex. Further, we confirm that astrocytes derived from hMMP-1 Tg animals secrete active hMMP-1. Taken together, these results support a critical role for PAR1 in synaptic transmission because dendritic patterning is one determinant of the amount of innervation that a neuron receives^8^.

Emerging evidence also suggests that MMPs are important modulators of dendritic spine number and structure. Spines are membranous protrusions that extend from the dendritic shaft and are the major sites of excitatory synaptic transmission. Inhibition of the gelatinase, MMP-9, corrects the immature dendritic spine phenotype found in in murine models of the autism-related syndrome, fragile X (FXS)^56^,^57^,^58^. Similarly, oral administration of minocycline, a broad-spectrum MMP inhibitor, to FXS animals promotes a shift in the spine profile from more immature to mature^59^. In agreement with a role for MMPs in spine remodeling, we find an increase in dendritic spine density in hippocampal CA1 pyramidal neurons. Given that astrocyte activation is observed with autism and FXS^16^,^60^, it is tempting to speculate that astrocyte derived proteases contribute to altered neuroplasticity in these conditions and more specifically, that the MMP-1/PAR1 signaling axis could represent a therapeutic target.

Changes in dendrite morphology and spine density impact the number of synaptic connections made on a neuron, which can influence learning and memory. Thus, MMP mediated structural changes could have consequences for behaviors affected by synaptic plasticity. Consistent with this concept, both protein and mRNA transcripts of MMP-3 and -9 are elevated during acquisition trials of Morris water maze, a spatial-hippocampal learning and memory task^7^. Further, MMP activity is critical to varied forms of hippocampal LTP^6^,^61^,^62^. Previous studies of MMPs in the setting of learning and memory have focused on family members that generate integrin binding ligands, which include MMP-2, -3, and -9^6^,^61^. Though neuronal activity dependent release of PAR1 activating MMP-1 or -13 has not been well studied, it is important to note that PAR1 signaling can enhance integrin avidity^63^. This leads us to speculate that PAR-activating MMPs may have additive or synergistic effects with those that generate specific integrin binding ligands.

Interestingly, however, the ability of MMPs to positively influence LTP appears to be a tightly regulated process: too much or too little MMP activity is inimical to the maintenance of LTP^1^. Here, we too provide data that overexpression of single MMP-1, which is observed in pathological conditions^13^,^15^, impairs performance in the Morris water maze. Two other behaviors are disrupted as well: decreased sociability and increased anxiety.

PAR1 is detected in neurons and astrocytes in hippocampus, cerebral cortex, and striatum of humans and mice with immunohistochemical techniques^19^,^20^,^21^. Electron microscopy studies in rat tissue also localize PAR1 to synaptic astrocyte endfeet^44^. Thus, PAR1 localization patterns suggest the receptor plays an important role in modulating plasticity. PARs belong to a unique 4-member family of GPCRs that are activated by site-specific proteolytic cleavage in the N-terminal extracellular region, which uncovers a tethered ligand that folds back onto the receptor conferring an active conformation^64^. Upon irreversible activation, the receptor is quickly internalized and degraded^37^ with sustained chronic activation leading to lower protein levels^38^. Consistent with these results, we too find decreases in PAR1 protein in lysates of mixed cortical/hippocampal regions in hMMP-1 Tg mice.

In terms of specific mechanisms by which PAR1 signaling affects neuronal structure, much of what we know about the role PAR1 plays in synaptic plasticity comes from studies that use thrombin, a potent peripheral activator that gains entry into the brain after blood brain barrier disruption and neurotoxicity^65^. It appears that thrombin induced PAR1 activation in the hippocampus and CNS cell types modulates synaptic transmission and plasticity through the enhancement of NMDA receptor currents and Ca^2+^ flux^66^,^67^. With respect to the study of MMP-1 as an agonist, it is important to note that PAR1 cleavage by thrombin and MMP-1 occurs at distinct locations in the N-terminus. Moreover, evidence suggests that thrombin and MMP-1-generated activating peptides differentially bias receptor signaling. In endothelial cells, MMP-1 and thrombin induce expression of different groups of pro-angiogenic genes through PAR1^68^. Once activated, PAR1 can signal through several G proteins including Gα_q/11_, Gα_i/o_, or Gα_12/13_ in addition to β-arrestin-mediated endosomal signaling^69^. Biased intracellular signaling depends on the activating ligand, availability of G proteins within the cell-type, and heterodimerization with other family members^70^. Interestingly, protease concentration can have opposing actions: high concentrations induce cell death whereas low doses appear to be neuroprotective^71^,^72^. What has yet to be determined is whether astrocyte-derived protease activation of PAR1 might also selectively activate this receptor in a biased manner. This will be investigated in future studies.

We find evidence supporting the selective activation of non-canonical G-protein independent signaling. Once activated, GPCRs are quickly targeted for internalization through phosphorylation of the C-terminus, which permits scaffolding proteins, such as β-arrestins, to bind and initiate internalization through endocytic complexes. In addition to its role in receptor desensitization, β-arrestin promotes G-protein independent signaling. Work focused on non-canonical signaling of dopamine receptors suggests that D2 receptor activation triggers the formation of a signaling complex through of β-arrestin 2, protein phosphatases, and Akt. This signaling pathway results in dephosphorylation of Akt and GSK-3β, which activates the latter^73^. We find evidence of selective activation of this pathway in hMMP-1 Tg animals as hippocampal lysates have increased levels of activated GSK-3β. High levels of active GSK-3β are linked to an increase in the number of thin spines in the dentate gyrus^41^. Additionally, using a broad spectrum MMP inhibitor the same study reversed the increased spine phenotype in constitutively active GSK-3β mice^41^.

In summary, we show that a protease driven by the expression of GFAP can influence neuronal plasticity through an ECM independent mechanism. These findings add to an emerging appreciation of glial derived factors as important effectors of brain structure and function, and have important implications for neurological disorders. Though outside the scope of the present study, future work will focus on sorting out the role of cell type specific contributions of PAR1 signaling and determining whether cell morphology changes are Ca^2+^-dependent. Lastly, identification of astrocyte derived synaptogenic factors and their mechanism of action provide the field with a more complete view of the complex interactions between neurons and glia during synaptic plasticity.

## Materials and Methods

### Animals

Experiments were conducted in accordance with the ethical guidelines of the National Institutes of Health and approved by the Institutional Animal Care and Use Committee at Georgetown University. All animals were group housed with littermates and permitted free access to food and water. Experimental results compare transgenic with littermates backcrossed to C57BL/6J (The Jackson Laboratory, stock #000664; http://jaxmice.jax.org/strain/000664.html) for at least 10 generations, unless stated otherwise.

### Preparation of primary cultures enriched for neurons

Cells were derived from WT and hMMP-1 Tg post-natal day 0 mouse pups. After the removal of adherent meninges, the hippocampi were removed and dissociated into a single cell suspension with trituration following incubation for 5 minutes with 0.1% trypsin as outlined in ref. 74. Dissociated cells were plated onto 18-mm sterilized glass coverslips or plastic 12-well culture dishes. Cultures were maintained in neural basal media supplemented with B27, glutamine, and penicillin/streptomycin antibiotic and incubated at 37 °C in a humidified 5% CO_2_-containing atmosphere. The neuronal enriched cultures also contained astrocytes as confirmed with GFAP and MAP2 immunocytochemistry (Anti-GFAP Millipore 5804 and Anti-MAP2 PhosphoSolutions, Aurora, CO).

### Dendritic Sholl analysis

Primary neuron-enriched cultures were treated with 8 nM human recombinant MMP-1 protein (hRecMMP-1) at DIV5 and DIV14. Cultures were fixed for 15 minutes with a 4% paraformaldehyde/sucrose solution two-hours after the second treatment at DIV14. Immunofluorescent techniques were used to label MAP2 cytoskeleton of neurons as previously described in ref. 75. Briefly cells were washed three times in PBS, permeabilized in PBS containing 2% (vol/vol) Triton X-100 (Sigma) for 10 min at RT followed by a 2-hour incubation in blocking solution (PBS containing 2% (vol/vol) Triton X-100 (Sigma) and 10% (vol/vol) goat serum). The primary antibody against MAP2 (chicken, 1: 2000, Phosphosolutions, Aurora, CO) was incubated overnight at 4 °C in a PBS antibody solution containing 1% (vol/vol) goat serum. On the second day, Alexa Fluor 488 conjugated goat anti-chicken secondary antibody (1: 2000, Invitrogen) was incubated at RT for 2 hours. Nuclei were counterstained with DAPI (1:10000, Sigma). Fluorescent mounting medium (Electron Microscopy Sciences, Hatfield, PA) was applied to slides as antifading agent prior to addition of coverslips. Images were acquired on Axioplan 2 Zeiss microscope. Next, we employed the use of a semi-automated Sholl analysis^27^. Briefly, 8-bit images of hippocampal neurons were traced using the NeuronJ plugin for ImageJ (NIH, Bethesda, MD) and then checked for accuracy in NeuronStudio^76^. Next, digitized images were analyzed in MATLAB using the open source Bonfire program (http://firesteinlab.cbn.rutgers.edu/downloads.html) to generate Sholl profiles. Researchers were blinded to treatment and genotype during image acquisition and analyses.

### Ca^2+^ Imaging

To investigate elevations in intracellular Ca^2+^, primary cultures enriched for neurons were loaded with a fluorescent Ca^2+^ indicator (Fluo-4 AM, 4 μM, Invitrogen) for 35 minutes at room temperature in physiologic buffer solution (140 mM NaCl, 4 mM KCl, 10 mM HEPES, 5 mM glucose, 2 mM CaCl_2_, and 1 mM MgCl_2_ at a pH of 7.3) followed by 5 buffer washes^77^. The dye was excited at 480 ± 15 nM. Emitted fluorescence was filtered with a 535 ± 25 bandpass filter and captured in real-time with Qimaging camera (Retiga 3000 M), which was read onto a neighboring computer. Analysis was sorted and performed offline using MATLAB and Simple PCI software (Compix Inc., Sewickley, PA, USA). The open source FluorSNNAP MATLAB code^78^ (http://www.seas.upenn.edu/~molneuro/fluorosnnap.html) was used to generate values for change in fluorescence above baseline (ΔF/F_0_).

### Generation of hMMP-1 transgenic animals

Transgenic animals were engineered to restrict human matrix metalloproteinase 1 (hMMP-1) expression to glial fibrillary acidic protein (GFAP) positive cells. Animals were generated by the transgenic core facility at Johns Hopkins University via pronuclear injection. Specifically, hMMP-1 cDNA (gift of Dr. J. D’Armiento, Columbia University) was subcloned downstream of the GFAP promoter (plasmid containing promoter sequence gift of Dr. M. Brenner, University of Alabama and subsequently injected into a B6SJF1 pseudopregnant female. Genomic PCR confirmed the presence of the transgene in the founder. PCR products were sequenced and matched against NCBI’s database. Resultant transgenic animals have been backcrossed to the C57BL/6J line for more than 10 generations.

### Genomic PCR

Genomic PCR for hMMP-1 transgene was performed on DNA samples isolated from mouse-tail biopsies collected upon weaning at post-natal day 21. DNA was purified using a traditional phenol-chloroform extraction protocol. The following primer sequences were used (forward: 5′AGC ACA TGA CTT TCC TGG AAT TGG C and reverse: 5′ATT TTG TGT TAG AAG AGT TAT CC). Tail biopsies were also sent to Transnetyx, Inc. (Cordova, TN) for genotyping.

### Preparation of primary astrocytes

Astrocytes derived from WT and hMMP-1 Tg mouse pups were prepared at post-natal day 1–2 as previously reported by our laboratory^79^. Specifically, after removal of adherent meninges, cortices were microdissected, incubated for 5 minutes with 0.1% trypsin, and dissociated into a single cell suspension with trituration. Dissociated cells were plated onto 18-mm sterilized glass coverslips or plastic 12-well culture dishes. Cells were maintained in MEM complete with Earle’s salts and L-glutamine (Gibco, catalog #11095) supplemented with 10% heat inactivated fetal bovine serum and penicillin-streptomycin. Cultures were maintained at 37 °C in a humidified 5% CO_2_-containing atmosphere. GFAP immunocytochemistry confirmed the presence of astrocytes in the cultures (≥95% of total cells were GFAP positive; anti-GFAP antibody, Millipore, 5804 or Cell Signaling, 3670 at a 1:1000-dilution).

### Western blot

Brains were dissected and lysed in RIPA buffer (50 mM Tris, pH 7.5, 150 mM NaCl, 0.1% SDS, 1% NP-40, and 1X protease and phosphatase cocktail (Thermo Scientific 1861281)). Lysates were sonicated for 10 seconds, placed on ice for 20 minutes, and centrifuged for 15 minutes at 14,000 rpm in 4 °C. Supernatants were recovered and used for future western blotting experiments. Protein concentrations were determined using BCA protein assay (Pierce Biotechnology, Inc.) and equal amounts of protein were used in all subsequent assays. Supernatants were incubated with Laemmli sample buffer (Bio-Rad, Hercules, CA, USA, catalog #161-0737) containing 5% β-mercaptoethanol and boiled for 5 minutes at 95 °C before denaturing electrophoresis on tris-glycine polyacrylamide gradient gels (Bio-Rad, Hercules, CA, USA). Proteins were transferred to nitrocellulose membranes, blocked in phosphate-buffered saline (PBS) containing 5% non-fat dry milk and 0.1% Tween (PBST) for 1 hour and subsequently probed with primary antibody at 4 °C overnight (PAR1: Santa Cruz H-111 at a 1:100-dilution). The following day membranes were washed 3 times for 15 minutes in PBST, species specific HRP-conjugated secondary antibody was applied at a 1:1000-dilution for 2 hours at room temperature, and followed by a second set of washes immunoreactive bands were visualized on film after incubation with chemiluminescence reagents (Perkin Elmer, NEL602).

### Enzyme linked immunosorbent assay (ELISA)

hMMP-1, phosphoGSK-3β, and total GSK-3β protein concentrations in mouse brain tissue were quantified by ELISA. All measurements were performed on lysates according to the manufacturer’s protocol (R&D systems, DMP100; Life Technologies, EMS2GSK3P and EMSGSK3T).

### RNAscope Fluorescent *in situ* hybridization combined with immunohistochemistry

hMMP-1 Tg mice and WT littermate controls were sacrificed and their brains were removed, fixed in 4% formol, embedded in paraffin and then sliced into 10-μm-thick sections and mounted onto Super Frost Plus slides (Fisher). FISH was performed according to the RNAscope 2.0 Red Fluorescent kit for formalin-fixed, paraffin-embedded brain sections (Advanced Cell Diagnostics (ACD)) according to the manufacturer´s instructions. Brain sections were dehydrated by 50%, 70%, and 100% ethanol gradually for 5 minutes, treated with pretreatment 2 solution during 15 minutes, and incubated for hMMP-1 probe (accession number: NM_001145938.1, manufactured by ACD) for 2 hours at 40 °C in the HybEZ humidified incubator. Following probe hybridization, brain sections experienced sequentially a series of probe signal amplification steps, rinsed in ACD Wash Buffer (2 × 2 minutes) and incubated in reagents after fluorescently labeled probes designed to target the red channel associated with hMMP-1 mRNA. Brain sections were washed in PBS (3 × 5 minutes) and blocked using Normal Goat Serum during 1 hour. Immunohistochemistry was performed using a primary antibody against rabbit anti-GFAP (Dako, 1:200) incubated overnight. Alexa Fluor 488-conjugated goat anti-rabbit IgG was applied for 2 hour (1:500) and slides were washed three times with PBS, counterstained with DAPI, and coverslips were mounted with Fluorogel with Tris Buffer mounting medium (Electron Microscopy Sciences). Images were obtained on an Axioplan 2 Zeiss microscope.

### MMP-1 activity assay

MMP-1 activity was tested using a fluorometric assay (AnaSpec) with a 60-minute endpoint reading. Prior to testing, supernatant samples were concentrated 40 times using 3 kDa cutoff centrifugal filters (VWR). The assay was performed according to the manufacturer’s instructions except that the 4-aminophenylmercuric acetate (APMA) sample activation step was omitted to permit measurement of endogenous secreted hMMP-1 activity. The standard curve was generated using recombinant human MMP-1 (R & D systems) and was linear within its range (0–8 ng).

### *In vivo* magnetic resonance imaging and spectroscopy

Magnetic resonance imaging (MRI) was performed at the Preclinical Imaging Research Laboratory of the Lombardi Comprehensive Center at Georgetown University Medical Center on a 7.0 Tesla Bruker horizontal bore Magnetic Resonance Imager run by Paravision 5.0 software as previously described^80^. Mice were anesthetized using 1.5% isoflurane and 30% nitrous oxide, positioned in a custom-made mouse stereotaxic device with temperature and respiration control and imaged in a 23 mm mouse volume coil. Two-dimensional MR anatomical locator images were acquired with a T2-weighted RARE protocol with the following parameters: TR: 3000 ms, TE: 24 ms, FOV: 2.25 cm, Matrix: 256 × 256, Averages: 4, Slice thickness: 0.5 mm. Magnetic resonance spectroscopy (MRS) allowed for the quantification of metabolic biomarkers. We used single voxel proton MRS with volume-localized PRESS sequence with the following parameters: TE: 20 ms, TR: 2500 ms, averages: 1024, spectral width of 4 kHz, and 512 k complex data points and 6 Hz line broadening, using a voxel of 2 mm on edge. The voxel was localized on the hippocampus and cortex based on the previously obtained locator image. All *in vivo* peak integrated areas were analyzed by visual inspection using the using the LC Model (see http://www.s-provencher.com/pages/lcmodel.html) software.

### Golgi staining and dendritic spine analysis

Golgi staining was performed on hMMP-1 Tg animals and WT littermate controls aged 3–4 months old using FD Rapid GolgiStain kit (FD NeuroTechnologies, Inc.; Columbia, Maryland) according to the manufacturer’s instructions. The brains were sliced on a vibratome (VT1000S; Leica) at 150 μm. Images of CA1 pyramidal neurons were taken in bright-field on an Axioplan2 Zeiss microscope at 63X or 100X. Images were coded, and dendritic spines counted in a blinded manner similar to previous protocols^81^. Dendritic branching was assessed in a blinded manner according to previously described methods^82^. Fully impregnated cerebral somatosensory cortical layer IV/V neurons were selected. Using a Zeiss Axioplan 2 microscope at 40X, a blinded investigator imaged multiple focal planes so that each basilar branch could be followed in its entirety. For each animal, a total of 9 neurons were evaluated to determine the number of primary, secondary, tertiary and quaternary branches.

### Behavioral testing

Animals were housed in temperature-controlled rooms with a 12-hour light/dark cycle. Cages were changed weekly; care was taken not to test animals on the same day as when animal facility changed cages. To further minimize confounding results due to stress, the mice were handled for 3 consecutive days prior to the start of testing. For all studies, the experimenter was blinded to genotype. Mice were permitted a minimum 30-minute habituation period to the testing room, and testing was performed at the same time of day except where noted. All comparisons were made between male littermates. Animals were 3–5 months of age at the time of testing. Three-chambered sociability assay. The social approach assay was slightly modified from previously described protocols^83^. Testing occurred in a 3-chambered apparatus where animals were permitted free access to all chambers during testing. The test was divided into two phases: habituation and social preference. During the habituation phase test animals were placed in center of apparatus box that contained two identical clear, Plexiglass cylinders with multiple holes to allow for air exchange at each end chamber. Animals were monitored for 10 minutes. Next, a stimulus mouse (gonadectomized A/J mice, The Jackson Laboratory, stock #000646; http://jaxmice.jax.org/strain/000646.html) was placed in one cylinder. The time spent in the social or non-social chamber was recorded over a 10-minute testing period. Analysis was performed with ANY-Maze (San Diego Instruments, San Diego, CA). Elevated plus maze. The elevated plus maze was performed by placing mice in the center of the apparatus and subsequently monitoring their movements over the course of 5 minutes. The plus shaped elevated platform consisted of two closed quadrants (wall height: 12 inches) and two open quadrants (no protective wall). Analysis was performed with ANY-Maze (San Diego Instruments, San Diego, CA). Morris water maze. Hippocampal learning and memory deficits were evaluated using the Morris water maze paradigm as previously described (Washington *et al*. 2012), with additional modifications. Specifically, the water maze apparatus consisted of a 4-foot-diameter pool (San Diego Instruments, San Diego, CA) filled with opaque water (25 °C; colored with non-toxic Crayola^®^ white paint). Visual cues were placed on the walls surrounding the pool and a platform (4 inches in diameter) was hidden below the surface of the water (1 cm). Initial training consisted of four trials per day over four days. Mice were introduced into the pool at variable entry points, with every entry point used over the course of the day. The location of the platform remained constant throughout training period. The mice were given 60 seconds to locate platform. On the fifth day of testing, a probe trial was conducted in which the platform was removed over one 60-second trial. Tracking software (ANY-Maze; San Diego Instruments, San Diego, CA) was used to record swim speed, total distance traveled, distance traveled in platform quadrant, time spent in platform quadrant, and platform crossings.

### Statistical analyses

Statistics were performed using Prism 5.0 (GraphPad Software). Individual statistical tests are listed in the figure legends as well as the number of samples. A *p* value ≤ 0.05 was considered significant and the following structure was applied to the figures in this paper ****≤*p* value 0.0001, ***≤*p* value 0.001, **≤*p* value 0.01, and *≤*p* value 0.05. Bonferroni’s, Dunnett’s or Tukey’s *post hoc* tests were performed when appropriate to correct for multiple hypothesis testing.

## Additional Information

**How to cite this article**: Allen, M. *et al*. Protease induced plasticity: matrix metalloproteinase-1 promotes neurostructural changes through activation of protease activated receptor 1. *Sci. Rep.* **6**, 35497; doi: 10.1038/srep35497 (2016).

## Supplementary Material

Supplementary Information
