# Supplementary material for: Protease induced plasticity: matrix metalloproteinase-1 promotes neurostructural changes through activation of protease activated receptor 1
Source: Sci Rep. 2016 Oct 20;6:35497. doi: 10.1038/srep35497 (PMC5071868; doi:10.1038/srep35497)
Supplement: Supplementary Information [file srep35497-s1.doc]

Protease induced plasticity: matrix metalloproteinase-1 promotes neurostructural changes through activation of protease-activated receptor-1

Megan Allen^1,2^, Suhasini Ghosh^2^, Gerard P. Ahern^2,3^, Sonia Villapol^2^, Kathleen A. Maguire-Zeiss^1,2^, and Katherine Conant^1,2,*^

^1^ Interdisciplinary program in Neuroscience, Georgetown University, Washington, DC

^2^ Neuroscience department, Georgetown University Medical Center, Washington, DC

^3^ Pharmacology and Physiology department, Georgetown University Medical Center, Washington, DC

 * Address correspondence to Dr. Katherine Conant (kec84@georgetown.edu)

Supplementary table 1. Quanitative Real time PCR results show that functionally similar MMPs are not detectable or changed in WT and hMMP-1 Tg animals.


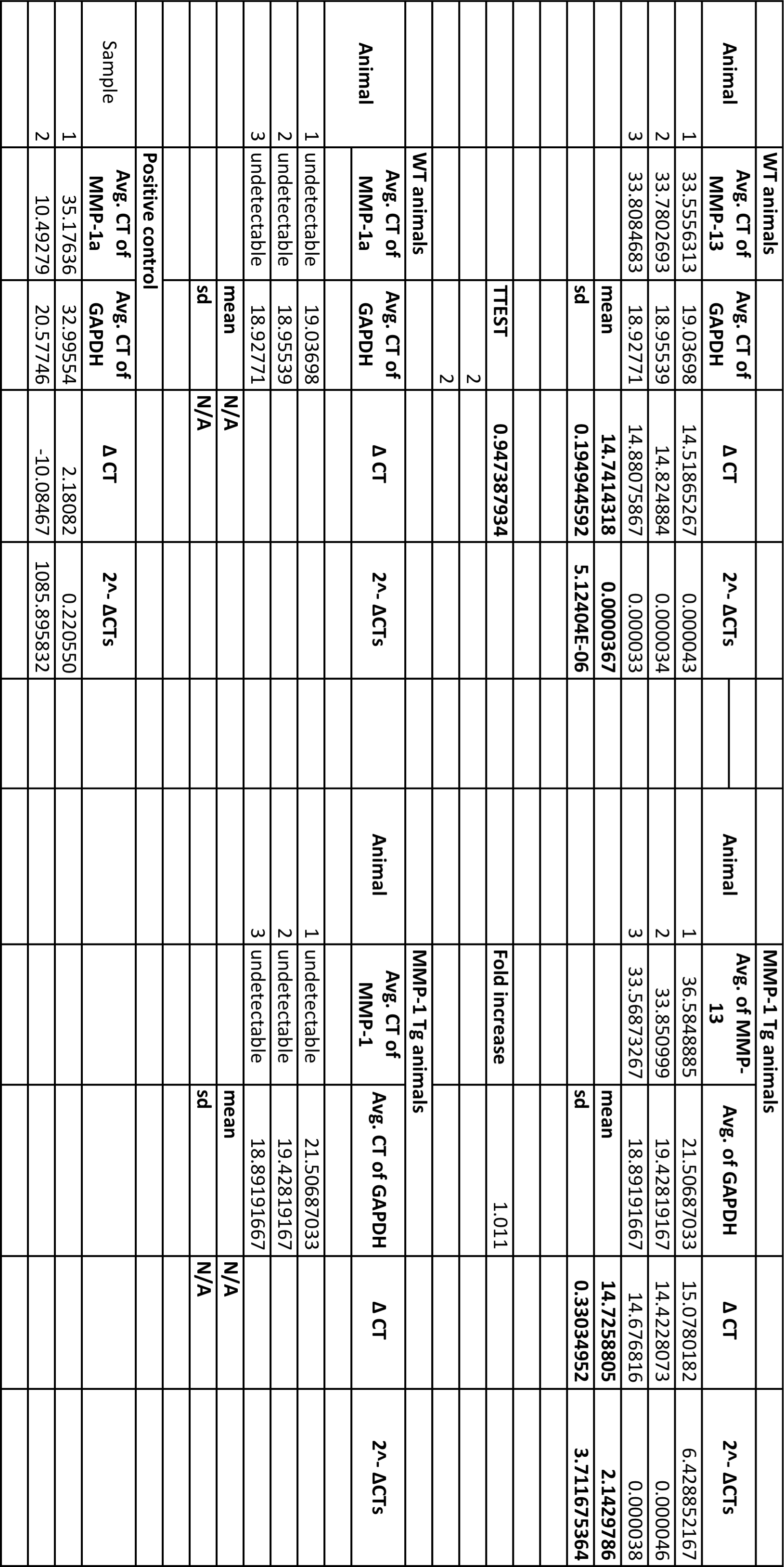


Supplementary materials and methods *Quantitative Real-Time PCR.* For measurements of gene expression in brain tissues, total RNA was isolated using Trizol (Invitrogen) followed by on-column DNase digestion with Rnase-free DNase (Qiagen, catalog # 79254). One μg of total RNA was reverse transcribed with random priming using MultiScribe MuLV reverse transcriptase (Applied Biosystems, catalog # 4368814). Quantitative real-time PCR was performed on 10 ng of the resulting cDNA as per the manufacturer’s specifications (Applied Biosystems). Primers were supplied by Life Technologies: *Mmp1a*: assay ID Mm00473485_m1, *Mmp13*: assay ID Mm01168714_m1, and *Gapdh*: assay ID Mm99999915_g1). Data were analyzed utilizing the relative quantification ΔΔCt method, normalizing target gene expression to a GAPDH endogenous control. Gene expression changes are represented as fold change (2^- ΔΔCt^). Statistical analyses were performed on ΔCt values as specified in the figure legends and the significance level was set at *P* < 0.05 ^1^.

Bibliography

1 Beraud, D. & Maguire-Zeiss, K. A. Misfolded alpha-synuclein and Toll-like receptors: therapeutic targets for Parkinson's disease. *Parkinsonism Relat Disord* **18 Suppl 1**, S17-20, doi:10.1016/S1353-8020(11)70008-6 (2012).
